# Peptic Ulcer Diseases: Genetics, Mechanism, and Therapies

**DOI:** 10.1155/2014/898349

**Published:** 2014-12-28

**Authors:** Seng-Kee Chuah, Deng-Chyang Wu, Hidekazu Suzuki, Khean-Lee Goh, John Kao, Jian-Lin Ren

**Affiliations:** ^1^Division of Hepato-Gastroenterology, Department of Internal Medicine, Kaohsiung Chang Gung Memorial Hospital and Chang Gung University College of Medicine, 123 Ta-Pei Road, Niao-Sung District, Kaohsiung City 833, Taiwan; ^2^Division of Gastroenterology, Department of Internal Medicine, Kaohsiung Medical University Hospital and Kaohsiung Medical University, Kaohsiung City 807, Taiwan; ^3^Division of Gastroenterology, National Hospital Organization Tokyo Medical Center, 2-5-1 Higashigaoka, Meguro-ku, Tokyo 152-8902, Japan; ^4^Department of Medicine, University of Malaysia, 50603 Kuala Lumpur, Malaysia; ^5^Department of Internal Medicine, Division of Gastroenterology, University of Michigan, 1150 W Medical Center Drive, 6520A MSRB 1, SPC 5682, Ann Arbor, MI 48109-5682, USA; ^6^Division of Gastroenterology, Zhongshan Hospital, Xiamen University, 201 Hubin South Road, Xiamen, Fujian 361004, China

Peptic ulcer disease is a very common disease which is mainly relevant to* Helicobacter pylori (H. pylori)* and nonsteroid anti-inflammatory drugs (NSAIDs) [1, 2]. Recent advances in biology and medicine have introduced new technologies to study the genetics of and the mechanisms underlying its pathology. Knowledge and understanding of these conditions have led to the development of animal models, successful therapies, and novel tools to characterize these clinical conditions and provide better care to patients. In this special issue, we invite investigators to contribute original research articles as well as review articles that will stimulate the continuing efforts to understand the underlying molecular issue, the development of strategies to treat these conditions, and the evaluation of outcomes. We are particularly interested in articles describing the new modalities for clinical characterization of this disease and measuring outcomes from treatment trials, advances in molecular genetics and molecular diagnostics, and current concepts in the treatment issues such as (1) recent genetic developments in peptic ulcer disease research such as genetic polymorphism and peptic ulcer disease, (2) recent advances in genetics and treatment of* H. pylori*, (3) latest technologies for clinical evaluation and measuring outcomes of peptic ulcer disease, (4) peptic ulcer disease mechanism using model systems such as* H. pylori*, (5) recent advances in peptic ulcer disease bleeding, (6) recent advances in peptic ulcer disease perforation and stenosis, and (7) recent advances in the relevant motility issue. Eventually, we published 11 papers overall.

Upper gastrointestinal bleeding (UGIB) guidelines improve patient care and outcomes [3–5]. This issue highlights the paper entitled “*Consensus on control of risky nonvariceal upper gastrointestinal bleeding in taiwan with national health insurance*” which highlighted the consensus report of Taiwan UGIB consensus meeting. It comprised recommendations from a nationwide scale to improve the control of UGIB, especially for the high-risk comorbidity group. The consensus included 17 statements, including 3 on preendoscopy, 5 on endoscopy, 6 on postendoscopy assessment, and 3 on Taiwan NHIRD regarding UGIB. The consensus highlighted that patients with comorbidities, including liver cirrhosis, end-stage renal disease, probable chronic obstructive pulmonary disease, and diabetes, are at high risk of peptic ulcer bleeding and rebleeding. Special considerations are recommended for such risky patients, including raising hematocrit to 30% in uremia or acute myocardial infarction, aggressive acid secretory control in high Rockall scores, monitoring delayed rebleeding in uremia or cirrhosis, considering cycloxygenase-2 inhibitors plus proton-pump inhibitors (PPI) for pain control, and early resumption of antiplatelets plus PPI in coronary artery disease or stroke. The consensus comprises practical recommendations to improve patient care of UGIB, especially for those with comorbidities. The most essential point of the Taiwan consensus is that it focuses more on comorbid patients. This is very important because peptic ulcer bleeding in comorbid patients is an emerging issue [6–8].

Low-dose aspirin is widely used in the prevention of cardiovascular disorders [9]. The genetic factors predicting the development of peptic ulcer in low-dose aspirin users remain unclear. The paper entitled “*Impact of blood type, functional polymorphism (T-1676C) of the COX-1 gene promoter and clinical factors on the development of peptic ulcer during cardiovascular prophylaxis with low-dose aspirin*” clarified that the C-1676T polymorphism in the* COX-1* gene promoter is not a risk factor for ulcer formation during treatment with low-dose aspirin. Blood type O, advanced age, history of peptic ulcer, and concomitant use of NSAID are of independent significance in predicting peptic ulcer development during treatment with low-dose aspirin.

Warfarin is currently the most commonly used oral anticoagulant worldwide with a narrow therapeutic window, wide variability in dose response across individuals, and a significant number of drug and dietary interactions and requires close laboratory monitoring with frequent dose adjustment [10, 11]. Gastrointestinal bleeding (GIB) is one of the severe bleeding complications of warfarin anticoagulation and occurs in up to 12% of cases [12]. The paper entitled “*Gastrointestinal hemorrhage in warfarin anticoagulated patients: incidence, risk factor, management, and outcome*” reported that warfarin was associated with a significant incidence of GIB in Taiwanese patients. The incidence of GIB was 3.9% per patient-years. Multivariate analysis with Cox regression showed that age >65 years old, a mean international normalized ratio >2.1, a history of GIB, and cirrhosis were independent factors predicting GIB. 27.3% of the GIB patients had rebleeding after restarting warfarin while thromboembolic events were found in 16.7% of the patients discontinuing warfarin therapy.

Reports regarding outcomes for different management regimens for peptic ulcer bleeding patients during holidays are inconsistent. Some described increased adverse outcomes on holidays [13, 14] while others did not [15, 16]. The paper entitled “*Outcome of holiday and nonholiday admission patients with acute peptic ulcer bleeding: a real-world report from Southern Taiwan*” observed that patients who presented with peptic ulcer bleeding on holidays did not experience delayed endoscopy or increased adverse outcomes. In fact, patients who received endoscopic hemostasis on the holiday had shorter waiting times, needed less transfused blood, switched to oral PPIs quicker, and experienced shorter hospital stays.

The paper entitled “*Comparison of hemostatic efficacy of argon plasma coagulation with and without distilled water injection in treating high-risk bleeding ulcers*” observed that endoscopic therapy with argon plasma coagulation (APC) plus distilled water injection was no more effective than APC alone in treating high-risk bleeding ulcers, whereas combined therapy was potentially superior for patients with poor overall outcomes.

UGIB is the most frequently encountered complication of peptic ulcer disease.* H. pylori* infection and nonsteroidal anti-inflammatory drug (NSAID) administration are two independent risk factors for UGIB [17–19]. The paper entitled “*Diagnosis, treatment, and outcome in patients with bleeding peptic ulcers and Helicobacter pylori infections*” reviewed and elucidated the relationship between bleeding peptic ulcers and* H. pylori* infection from the chronological perspective with an emphasis on diagnosis, treatments, and outcomes. They summarized that sufficient evidence supports the concept that* H. pylori* infection eradication can heal the ulcer and reduce the likelihood of rebleeding. With increased awareness of the effects of* H. pylori* infection, the etiologies of bleeding peptic ulcers have shifted to NSAID use, old age, and disease comorbidity.

It is urgent to find alternative agents due to increasing failure rate of* H. pylori* eradication [20, 21]. The paper entitled “*Does long-term use of silver nanoparticles have persistent inhibitory effect on H. pylori based on mongolian gerbil's model?*” surveyed the long-term effect of silver nanoparticles (AgNP) on* H. pylori* based on Mongolian gerbil's model. They concluded that AgNP/clay would be a potential and safe agent for inhibiting* H. pylori*. It should be helpful for eradication of* H. pylori*.

*Helicobacter pylori* infection leads to chronic inflammation of gastric mucosa and peptic ulcer disease. It may influence the absorption of essential trace elements. The association between trace elements and* H. pylori* infection has been reported [22]. The paper entitled “*The effect of Helicobacter pylori eradication on the levels of essential trace elements*” is designed to compare the effects of* H. pylori* infection treatment on serum zinc, copper, and selenium levels. They concluded that* H. pylori* eradication regimen appears to influence the serum selenium concentration.

*H. pylori* were linked with several extragastrointestinal diseases, including preeclampsia and intrauterine growth restriction of fetus. There are several methods to detect* H. pylori* infection. One of them is the urease test using gastric mucosal tissue obtained during gastroendoscopy. Despite being proven that procedure is safe when performing on the pregnant women [23], the general unwillingness, the high cost, the invasiveness of the procedure, and the possible sampling error make it not the ideal choice for screening the* H. pylori* infection during pregnancy. The noninvasive tests include the urea breath test (UBT), the stool antigen test, and the serum* H. pylori* IgG antibody test. The latest one is easy to perform during antenatal examination and the existence of the antibody was found to be associated with the intrauterine growth restriction [24]. How the maternal* H. pylori* antibody influences the growth of the fetus is still elusive, but, interestingly, the antibody can be transmitted transplacentally to the fetus [25, 26]. However, the detection of the serological antibody was frustrated because of the inconsistent accuracy caused by several factors, including the different antigen extracts, the kit uses, and variable* H. pylori* strain in different regions [27, 28]. The paper entitled “*The utilization of a new immunochromatographic test in detection of Helicobacter pylori antibody from maternal and umbilical cord serum*” utilized a commercial immunochromatographic kit to detect the antibody in maternal and cord serum. The authors found out that* H. pylori* IgG antibody can be transferred through the placenta into the fetal circulation. However, accuracy of the test kit needs to be evaluated before utilization in screening.

Patients who have experienced severe caustic injury to the gastrointestinal tract are at high risk of luminal strictures [29]. Early endoscopy is usually routinely recommended in patients after gastroesophageal caustic injuries and should be performed to prevent unnecessary hospitalization and to plan future treatment after carefully assessing the severity of the initial digestive lesions [30]. The paper entitled “*Predicting the progress of caustic injury to complicated gastric outlet obstruction and esophageal stricture, using modified endoscopic mucosal injury grading scale*” indicates that patients over 60 years have a higher mortality rate after corrosive injury of gastrointestinal tract and, therefore, require attentive care in acute stage. And, early endoscopy to grade the extent of mucosal injury is useful to predict the incidence of subsequent stricture of GI tract and provide valuable information on clinical follow-up.

Gastrointestinal tract disorders are common in diabetic patients [31, 32]. More than 75% of patients visiting diabetes mellitus clinics reported significant gastrointestinal symptoms [31] such as dysphasia, early satiety, reflux, abdominal pain, nausea, vomiting, constipation, and diarrhea. In the paper entitled “*Decreased gastric motility in type II diabetic patients*,” the authors hypothesized that diabetic patients had lower motilin and ghrelin or higher glucagon-like peptide-1 (GLP-1) and hence inhibited gastric motility and induced gastrointestinal symptoms. They compared gastric motility and sensation between type II diabetic patients and normal controls and explored the roles of different gastric motility peptides in this motility effect. Type II diabetic patients have delayed gastric emptying and less antral contractions than normal controls and may be associated with less postprandial sensation. They concluded the observation that less serum GLP-1 in type II diabetic patients could offer a clue to understand that delayed gastric emptying in diabetic patients is not mainly regulated by GLP-1.

*Seng-Kee Chuah* *Deng-Chyang Wu* *Hidekazu Suzuki* *Khean-Lee Goh* *John Kao* *Jian-Lin Ren*
